# Drivers and Moderators of Rural-Urban Differences in Caregiving Intensity and Health-Related Quality of Life

**DOI:** 10.1093/geroni/igaf122.800

**Published:** 2025-12-31

**Authors:** Steven Cohen, Mary Greaney

**Affiliations:** University of Rhode Island, Kingston, Rhode Island, United States; University of Rhode Island Department of Health Studies, Kingston, Rhode Island, United States

## Abstract

Rural-urban disparities are pervasive among older adults and their caregivers. Rural areas in the US have less access to services and a disproportionately high population of older adults, and are more reliant on informal, family caregivers than those in urban and suburban areas. There is a need to understand critical, rural-urban differences in caregiving intensity and caregiver health-related quality of life (HRQoL) across the US. Data were abstracted and merged from the 2022 and 2023 Behavioral Risk Factor Surveillance System (n = 31,164), a nationally representative data set. Generalized linear models were used to assess associations between rural-urban status and caregiving intensity and HRQoL, and model associations between caregiving intensity and HRQoL, adjusting for demographics and social determinants of health and accounting for complex sampling. Rural caregivers had a significantly higher likelihood of providing >20 hours/week of care (odds ratio [OR] 1.10, 95%CI 1.01-1.19), reporting fair/poor general health (OR 1.30, 95%CI 1.19-1.42) and having >5 days/month of poor physical health (OR 1.16, 95%CI 1.07-1.26). However, rural caregivers had a lower likelihood of reporting >5 days/month of poor mental health (OR 0.88, 95%CI 0.82-0.96) and providing care for someone with Alzheimer’s disease/dementia (OR 0.87, 95%CI 0.77-0.98) than urban caregivers. There were no significant differences in associations between caregiving intensity and HRQoL by rural-urban status. The study identified notable rural-urban differences in caregiving intensity and aspects of HRQoL that likely impact the health and quality of life of informal caregivers, suggesting that policies and interventions to address caregiver needs must account for geographic context.

